# Roles of Small GTPase Rac1 in the Regulation of Actin Cytoskeleton during Dengue Virus Infection

**DOI:** 10.1371/journal.pntd.0000809

**Published:** 2010-08-31

**Authors:** Jia-Li Wang, Jun-Lei Zhang, Wei Chen, Xiao-Feng Xu, Na Gao, Dong-Ying Fan, Jing An

**Affiliations:** 1 Department of Microbiology, Third Military Medical University, Chongqing, People's Republic of China; 2 Department of Genetics, Third Military Medical University, Chongqing, People's Republic of China; 3 Department of Microbiology, School of Basic Medical Sciences, Capital Medical University, Beijing, People's Republic of China; Tropical Medicine Institute Pedro Kourí (IPK), Cuba

## Abstract

**Background:**

Increased vascular permeability is a hallmark feature in severe dengue virus (DV) infection, and dysfunction of endothelial cells has been speculated to contribute in the pathogenesis of dengue hemorrhagic fever/dengue shock syndrome (DHF/DSS). Rho-family GTPase Rac1 is a significant element of endothelial barrier function regulation and has been implicated in the regulation of actin remodeling and intercellular junction formation. Yet there is little evidence linking Rac1 GTPase to alteration in endothelial cell function induced by DV infection.

**Methods and Findings:**

Here, we showed that actin is essential for DV serotype 2 (DV2) entry into and release from ECV304 cells, and Rac1 signaling is involved these processes. At early infection, actin cytoskeleton rearranged significantly during 1 hour post infection, and disrupting actin filament dynamics with jasplakinolide or cytochalasin D reduced DV2 entry. DV2 entry induced reduction of Rac1 activity within 1 hour post infection. The expression of dominant-negative forms of Rac1 established that DV2 entry is negatively regulated by Rac1. At late infection, actin drugs also inhibited the DV2 release and induced accumulation of viral proteins in the cytoplasm. Meanwhile, the activity of Rac1 increased significantly with the progression of DV2 infection and was up-regulated in transfected cells expressing E protein. Confocal microscopy showed that DV2 E protein was closely associated with either actin or Rac1 in DV2-infected cells. The interaction between E protein and actin was further confirmed by co-immunoprecipitation assay.

**Conclusions:**

These results defined roles for actin integrity in DV2 entry and release, and indicated evidence for the participation of Rac1 signaling pathways in DV2-induced actin reorganizations and E-actin interaction. Our results may provide further insight into the pathogenesis of DHF/DSS.

## Author Summary

An important clinical characteristic of dengue hemorrhagic fever/dengue shock syndrome is increased vascular permeability. Actin cytoskeleton is a significant element of endothelial barrier function regulation. *In vitro* study showed that dengue virus infection could induce redistributions of actin cytoskeleton. It is not precisely clear the roles of actin and the mechanisms of its reorganization during the infection. Using immunochemical assays, drug inhibition assays and protein interaction profiling methods, we aimed to identify the ways in which dengue virus serotype 2 interacts with actin cytoskeleton. The study showed that dynamic treadmilling of actin is necessary for dengue virus entry, production and release, while small GTPase Rac1 also plays multiple roles during these processes. In addition, we demonstrated the association of viral E protein with actin, indicating a direct effect of viral protein on the structural modifications of actin cytoskeleton. Our results provide evidence for the participation of Rac1 signaling pathways in viral protein-induced actin reorganizations, which may be a mechanism involved in the etiology of dengue hemorrhagic fever.

## Introduction

Dengue virus (DV) is an enveloped, single-stranded RNA virus belonging to the family Flaviviridae. The DV genome has one open reading frame encoding three structural proteins - capsid, membrane and envelope (E)- that constitute the virus particle, and seven nonstructural proteins. DV infection causes a wide range of symptoms from a mild disease (dengue fever, DF) to severe, life-threatening complications (dengue hemorrhagic fever/dengue shock syndrome, DHF/DSS). The characteristics of DHF/DSS are abnormalities in hemostasis and increased vascular permeability. Sudden and extensive plasma leakage in tissue spaces and various serous cavities of the body in patients with DHF may result in profound shock – DSS – that can be fatal if not clinically managed in time [1]. However, the mechanism of the increased vascular permeability induced by DV infection is not clear yet. Autopsy studies showed rare apoptotic endothelial cells and no severely damaged capillaries vessels, though capillaries in several organs showed endothelial swelling [2]. It seemed that increased vascular permeability without morphological destruction of capillary endothelium is the cardinal feature of DHF/DSS [3].

Dynamics of cytoskeletal and cytoskeleton-associated proteins is a significant element of endothelial barrier function regulation. Actin cytoskeleton, linking to the cytoplasmic tail of junctional adhesive proteins as well as extracellular matrix protein, is relevant in the stabilization of inter-cellular junctions and the maintenance of endothelium integrity. In our previous study, increased permeability of monolayer of ECV304 cells without obvious morphological destruction was observed in DV2-infected cell culture model [4], and β3 integrin, which is an extracellular matrix protein and plays central roles in maintaining capillary integrity, showed an up-regulating expression in human dermal microvascular endothelial cells after DV2 infection [5]. Additionally, several groups also reported that DV infection induce alterations in actin cytoskeletal assembly and junctional protein complexes in human vascular endothelial cells in vitro [6]–[8]. Therefore, it was inferred that actin rearrangement induced by DV infection may contribute to the dysfunction of endothelial barrier, which in turn cause increase of vascular permeability.

Actin and the associated vesicle fission machinery act in concert to liberate nascent vesicles from both the plasma membrane and trans-Golgi network [9]. Recent work revealed that some viruses that enter via receptor-mediated endocytosis and bud at plasma or endosomal membrane recruit cellular actin cytoskeleton for these purposes [10], [11]. After virus infection, actin cytoskeleton may also interact with viral structural proteins, such as envelope protein of another flavivirus (West Nile virus), and the nucleocapsid protein of a hantavirus (Black Creek Canal virus) [12], [13]. As observed by ourselves and others, DV also enter host cells via clathrin-dependent endocytosis, and viral nucleocapsids are released into the extracellular by budding [14], [15]. It has been implicated that actin rearrangement is required for DV entry into the mosquito C6/36 cells and mammalian HepG2 cells [16], [17]. The components involved in regulating actin rearrangements of the infected cultural system remain to be identified.

Rac1 GTPase, a member of Rho GTPases family, has been implicated in the negative regulation of clathrin-mediated endocytosis [18]. Rac1 also has a central role in regulating both actin cytoskeletal remodeling and the integrity of intercellular junctions. In the absence of vasoactive stimuli, dominant negative Rac1 increases endothelial permeability and affects adherens and tight junctions. Recent studies showed Rac1 GTPase is essential for many virus infections. HIV Env-mediated syncytium formation relies on Rac1 activation in target cells [19]. Early herpes simplex virus type 1 infection is dependent on regulated Rac1 signalling in epithelial cells [20]. Rac1 activation is also required for hepatitis B virus replication and Coxsackievirus movement [21], [22]. A very recent study reported that Rac1 and Cdc42 regulates formation of filopodia required for DV2 entry into HMEC-1 cells [23]. However, current knowledge about the mechanism of actin organization induced by DV infection is still limited.

Here we sought to investigate the roles of Rac1 GTPase and the participation of viral E protein in actin rearrangements of ECV304 cells induced by DV2 infection. Our data showed that actin cytoskeleton is required for DV2 infection, during which Rac1 GTPase may play different roles at various stages. In the early stage, DV2 entry reduces activity of Rac1 GTPase and DV2 entry is negatively regulated by Rac1 GTPase. In contrast, Rac1 is activated by DV2 infection in the late stage and also by the expression of viral E protein. Confocal study indicated active Rac1 may be involved in the interaction between actin and E protein, and hence actin reorganizations. Taken together, these results suggested multiple roles of Rac1 GTPase in DV2 infection.

## Materials and Methods

### Cells and virus

*Aedes albopictus* mosquito (C6/36) cells and endothelial-like ECV 304 cells (European Collection of Cell Cultures) were grown in Dulbecco's Eagle's minimum essential medium (DMEM, GIBCO) containing 10% fetal bovine serum (FBS). Vero cells were grown in Eagle's minimum essential medium (MEM, GIBCO) with 5% FBS.

DV2 (strain Tr1751) virus was isolated from a patient with dengue fever and propagated in C6/36 cells and stored at −70°C until used. Viral titers were detected by plaque assay, using a Vero cell monolayer culture under 1% methylcellulose overlay medium.

### Antibodies and chemicals

Rabbit anti-E protein of DV2 polyclonal antibodies (PAb) and mouse anti DV2 PAb were produced in our laboratory. Mouse monoclonal antibodies (MAb) 301 and 504 against Japanese encephalitis virus [24],which cross-reacted with E protein of DV2, were kindly provided by Dr. Yasui K (Tokyo Metropolitan Institute for Neuroscience, Japan). FITC-conjugated goat anti-mouse immunoglubin (IgG), rabbit anti-actin PAb, anti-Rac1 MAb, phalloidin–TRITC, and protease inhibitor cocktail were from Sigma. Cytochalasin D (Cyt D), jasplakinolide (Jas), and 3-(4,5-dimethyl thiazol-2yl)-2,55-diphenyltetrazolium bromide (MTT) were from Merck. Lipofectamine reagents were from Invitrogen. pReceiver-M01α vector was from Stratgene. Glutathione sepharose 4B and anti GST antibody were from Amersham.

### Generation of ECV304 cells expressing DV2 E proteins

To attain the vector expressing DV2 E protein (GenBank accession number: L10053.1), the coding sequences of E genes (from 877 to 2421 bp, containing the gene coding signal peptides) were amplified and cloned into the Nsp5 and XhoΙ sites of pReceiver-M01α,named pRec-E. The sequences of PCR generated fragment was verified by DNA sequencing. Then ECV304 cells were transfected with plasmids pRec-E by using Lipofectamine 2000 according to the manufacturer's instruction. The distributions of recombinant DV2 E proteins with actin filaments were analyzed by immunofluorescence assay at 48 h post-transfection. To study the effect of E proteins on Rac1 activity, cells stably expressing pRec-E or pReceiver-M01α were obtained after selection under G418 (400 µg/ml) for 10 days and named as ECV/pRec-E and ECV/pRec respectively.

### Generation of stable ECV304 cells expressing Rac1-WT, Rac1-V12, and Rac1-N17

To attain the vectors expressing Rac1 mutants, the wild-type (WT) forms and constitutively-inactivated mutants of Rac1 were amplified from Rac1 cDNAs, pEXV3-Rac1V12N17 and pEXV3-Rac1N17 respectively [25], with sequence specific primers and cloned in the sense orientation into the Nsp5 and XhoΙ sites of pReceiver-M01α. The sequences of all PCR generated fragments were verified by DNA sequencing. ECV304 cells stably transfected with the His-tagged WT, V12N17, and N17 mutants of Rac1 plasmids were established by using Lipofectamine 2000 according to the manufacturer's instruction. And each cell line carrying either a WT or mutant Rac1 plasmid was first characterized by a GTP-loading assay to confirm its phenotype. These cell lines were used for studying effect of Rac1 on DV2 infection.

### Assay for Entry efficiency of DV2

Cells were inoculated with DV2 (MOI = 10) and incubated at 37°C for 1 h. Then the inoculum was removed and the cells were treated with acid glycine (pH 3.0) solution for 2 min to inactivate extracellular virus. Afterwards, cell samples were collected and the titer of virus in each sample was determined by standard plaque assays on Vero cells. The titer of mock treatment (DMSO control) was considered as 100%. Experiments were performed in duplicate for at least three independent experiments and the titers were averaged.

### Studies of the actin inhibitor effect on DV2 infection

Two drugs, Jas and Cyt D, were stored at −20°C as 1 mM or 100 µM stock solutions in dimethyl sulfoxide (DMSO) respectively. The cytotoxicity of each drug to ECV304 cells was determined, using the 3-(4,5-dimethylthiazol-2-yl)-diphenyl tetrazolium bromide (MTT, Sigma) method. From this, 100 nM of Jas and 4 µM of Cyt D were used as the highest working concentrations, and DMSO was added at the same concentration to the control cells (mock treatment).

To study the effects of actin inhibitor on DV2 entry, ECV304 cells were grown to confluence in 24-well culture plates and pretreated with DMEM containing Jas (100, 50, or 10 nM), Cyt D (4, 2, or 0.2 µM), or 0.1% of DMSO (mock-treatment) for 5, 3, or 1 h at 37°C, respectively. Then the infection was performed and the entry efficiency of DV2 was determined as described above.

To study the effects of actin inhibitors on virus yield, Cyt D or Jas was added to the infected cells at different time points after viral entry. ECV304 cells were grown to confluence in 6-well culture plates, and inoculated with DV2 (MOI  = 1) at 37°C for 1 h. Medium containing drugs (100 nM of Jas or 2 µM of Cyt D) was added to the cells at the initiation of infection, then the cells were fixed at 1 d or 3 d post infection (p.i.) and virus antigens were detected with immunofluorescence assay as described below. Medium containing drugs was also added at the indicated time points post infection (0 h p.i., 3 h p.i., or 6 h p.i. respectively) and left on for the entire duration of the 9-h incubation. Control cells were incubated with medium containing 0.1% of DMSO (mock-treatment). At 9 h p.i., 2 ml of supernatant was saved and cells were frozen and thawed three times in 2 ml of DMEM. Viral titer of each sample was determined in duplicate by plaque assay and plotted as percentages of the titer in the samples of control cells.

### Co-immunoprecipitation (co-IP) of DV2 E protein with actin

Infected or mock-infected ECV304 cells (approximately 5×10^6^) were rinsed in cold phosphate-buffered saline (PBS) and lysed in 500 µl of lysis buffer (10 mM Tris HCl [pH 7.5], 1 mM EDTA, 100 mM NaCl, 1% NP-40, 1% protease inhibitor cocktail) for 30 min on ice. After centrifugation at 12,000 rpm for 10 min, the clarified cell lysate was mixed with MAbs (mixture of 301 and 504) and rocked end-over-end at 4°C overnight. Subsequently, 20 µl of protein A-Sepharose beads (50% slurry) was added and incubated for 1 h at 4°C. The beads were collected and washed three times in the lysis buffer. The bound proteins were dissociated by boiling for 5 min in the Laemmli sample buffer and separated on 12% polyacrylamide gels.

### Expression and purification of GST-CRIB

CRIB was expressed in *E. coli* strain BL21 as a GST fusion protein and immobilized by binding to glutathione-Sepharose beads. Briefly, the cDNA sequence of CRIB domain was amplified from mouse brain and cloned into the pGEX-6p-1. Then, *E. coli* transformed with recombinant plasmids was grown at 37°C overnight in 2xYT medium. The next morning, the culture was diluted 1∶20 into fresh 2xYT medium and cells were induced with 0.5 mM ipTG (isopropyl-beta-D-thiogalactopyranoside), and growth was continued for 24 h at 25°C. The cells were harvested with centrifugation at 5000 g for 10 min, and resuspended in PBS (50 µl per ml of culture). Then the cells were lysed by sonication on ice, incubated on ice for 20 min in the presence of 1%Triton X-100. After centrifugation at 8000 g for 10 min, the soluble fraction was incubated with glutathione sepharose 4B at 4°C, with gentle agitation overnight. The beads were then washed three times with 10 ml PBS-1% Triton X-100 and two times with 10 ml PBS. The washed GST-CRIB beads were kept at 4°C in the presence of sodium azide and a cocktail of protease inhibitors.

### Pull-down assay for Rac1-GTP

Activated Rac1 was identified by binding specifically to the GST-fused p21-binding domain of human Pak1. Briefly, ECV304 cells (approximately 5×10^6^) were seeded and serum starved for about 24 h. After infection with DV2, cells were washed twice in ice-cold PBS and lysed in 500 µl of lysis buffer (10 mM Tris HCl [pH 7.5], 1 mM EDTA, 100 mM NaCl, 1% NP-40, 1% protease inhibitor cocktail) at various time points p.i. The lysates were clarified, normalized to equal amounts of total proteins, and incubated with glutathione beads containing bound GST-CRIB for 90 min at 4°C. Bound Rac1 was resolved by sodium dodecyl sulfate-12% polyacrylamide gel electrophoresis (SDS-12% PAGE) and immunoblotted with monoclonal antibodies against Rac1. Immunoreactive bands were visualized, and band intensities were assessed. The bands were scanned, and their intensities were assessed and quantified, with GTP bound Rac1 in mock-infected cells being considered one fold activation for comparison to infected cells.

### *In situ* detection of Rac1-GTP

A novel affinity binding assay has been developed for in situ detection of active form of small GTPases in mammalian cultured cells [26]. Briefly, bound GST-CRIB was eluted off the beads with elution buffer (50 mM Tris-HCl, 10 mM reduced glutathione, pH 8.0) and stored at −80°C before use. ECV304 cells were infected with DV2 ( MOI  = 1) for 24 h and then cells were fixed in 2% paraformaldehyde buffer on ice for 1 min and then incubated with GST-CRIB (100 µg/ml) on ice for 10 min. After washing, cells were fixed again in paraformaldehyde buffer on ice for 10 min. Subsequently, GST-CRIB was detected with an anti-GST antibody and a secondary antibody conjugated with horseradish peroxidase.

### Immunofluorescence analysis

Cells were fixed with 4% paraformaldehyde for 15 min and permeabilized with 0.2% Triton-X 100 for 5 min. After washing and blocking with 1% bovine serum albumin (BSA) in PBS, polymerized actin was detected by incubating the cells with phalloidin-TRITC (1∶100, 1 h at 37°C). For detections of actin filaments and DV2 antigens or E protein, cells were incubated with mouse anti-DV2 PAb (1∶100, overnight at 4°C) or with a mixture of MAb 301 and 504 (1∶100, overnight at 4°C), and subsequently with a mixture of FITC-conjugated goat anti-mouse IgG (1∶100) and phalloidin-TRITC (1∶100, 1 h at 37°C). For detections of Rac1 and E protein, cells were incubated with primary mouse anti-Rac1 MAb (1∶100, overnight at 4°C) and FITC-conjugated secondary antibody (1∶100, 1 h at 37°C). Then cells were incubated with rabbit anti-E PAb (1∶100, overnight at 4°C) and TRITC-conjugated secondary antibody (1∶100, 1 h at 37°C). Following washing with PBS, slides were mounted with mounting medium and examined under a fluorescent microscope (Olympus, BX-51) or a confocal laser microscope (Leika TCS-NT). The relative virus positive area was measured by Image Proplus 5.0 program and the area of control cells was considered as 100%.

### Statistical analysis

The statistical significance was accessed by two-tailed paired student's t-test.

## Results

### 1. Actin cytoskeleton is required for DV2 entry

**Figure 1:**
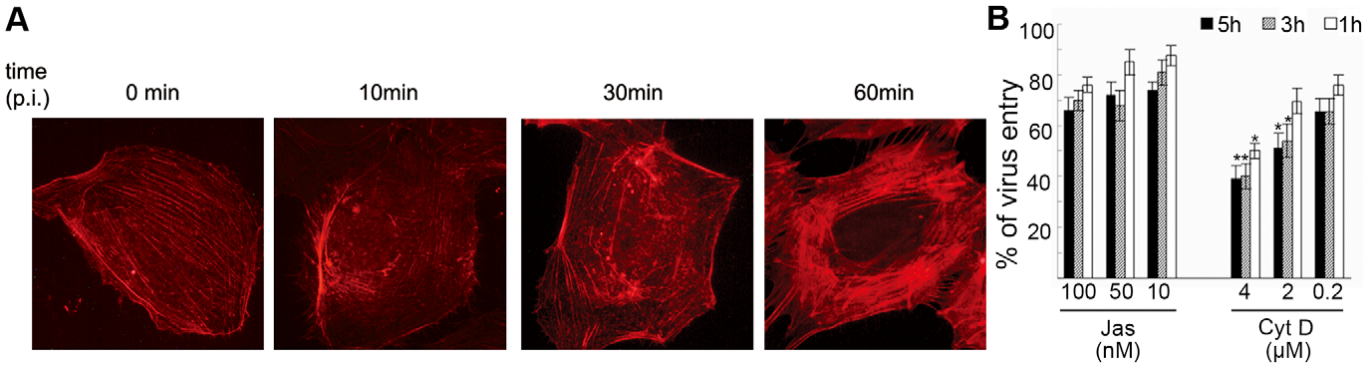
Actin is required for DV2 entry. A. DV2 infection induces rearrangements of the actin filaments in ECV304 cells. Serum-starved ECV304 cells were incubated with DV2 at 37°C. Then cells were fixed at the indicated time points, and stained for F-actin with phalloidin-TRITC. (×1600). B. Dose and time-dependent inhibition of DV2 entry by actin inhibitors. ECV304 cells were incubated with DMEM containing different concentrations of Jas (100, 50, or 10 nM) or Cyt D (4, 2, or 0.2 µM) for 1 h, 3 h, or 5 h. As a control, cells were pretreated with 0.1% DMSO, a solvent for these two inhibitors. The cells were infected with DV2 in the presence or absence of inhibitors at 37°C for 1 hour. Then the cells were treated with acid glycine solution (pH 3.0) for 2 min at room temperature to inactivate extracellular viruses, washed twice with PBS, and collected. The titers of cell samples were measured by plaque assay with Vero cells. The titer of mock treatment (DMSO control) was considered as 100%. Experiments were performed in duplicate for at least three independent experiments. *P<0.05 vs. DMSO control.

Actin cytoskeletal assembly/disassembly dynamics are critical for many aspects of clathrin-coated structure dynamics including assembly, constriction, internalization, and lateral motility [27]. Recent studies have shown that WNV and DV enter the mammalian cells in clathrin-mediated endocytosis, but direct functional evidence for a role of actin during DV2 entry is lacking. In this study, the addition of DV2 caused rapid reorganizations of F-actin network in ECV304 cells within 10 min p.i. (Fig. 1A). Actin fibers disassembled and dispersed drastically throughout the cytoplasm at 30 min p.i., then repolymerized into stress fibers along the cell edge at 1 h p.i., indicating that DV2 infection could induce reorganization of actin cytoskeleton.

To investigate the effect of the interrupted actin cytoskeleton on virus entry, ECV304 cells were pretreated with Jas or Cyt D, and then infected with DV2 at 37°C for 1 h. As shown in Fig. 1B, the virus entry into drug-treated cells was inhibited in a time and dose dependent manner when compared with that in mock treated cells. At 1 h of treatment, about 40%, 19%, and 15% reduction at 4, 2, 0.2 µM of Cyt D were induced, and there was no significant inhibition at 50 or 10 nM of Jas and a little reduction with about 13% at 100 nM of Jas. About 50%, 39%, and 27% reduction at 4, 2, 0.2 µM of Cyt D, and about 25%, 20%, and 17% reduction at 100, 50, 10 nM of Jas were observed at 5 h of treatment, respectively. These results showed that actin dynamics is essential for the DV2 entry.

### 2. Reduction of Rac1 GTPase activity is essential for DV2 entry

**Figure 2:**
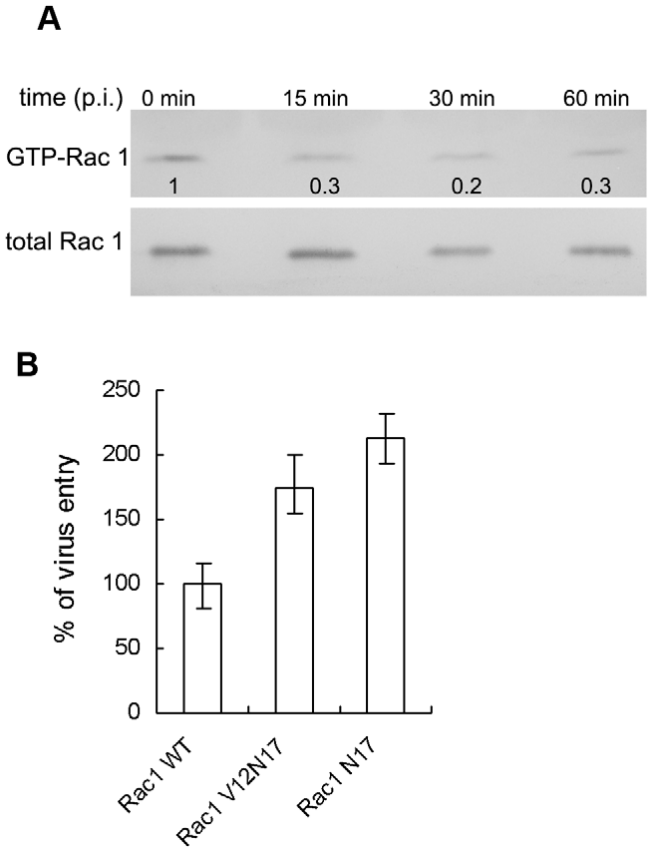
The role of Rac1 in DV2 entry. A. DV2 entry induces inactivation of Rac1 GTPase. ECV304 cells were serum starved for 24 h and infected with DV2. Equal amounts of cell lysates from mock-infected cells, or cells infected with DV2 at different time points as indicated were used to capture the GTP-bound forms of Rac1 GTPase by GST-CRIB pull down assays. The proteins captured were analyzed by SDS–12% PAGE and immunoblotted with anti-Rac1 (top panels). Normalized cell lysates were analyzed for total Rac1 as a loading control (bottom panels). B. Overexpression of dominant-negative Rac1 mutant proteins increases entry of DV2. ECV304 cells were stable transfected with plasmids encoding a wild-type (WT), or two dominant-negative forms (N17, V12N17) of Rac1. The cells were infected with DV2 at 37°C for 1 hour. Then the cells were treated with acid glycine solution (pH 3.0) for 2 min at room temperature to inactivate extracellular viruses, washed twice with PBS, and collected. The titers of cell samples were measured by plaque assay with Vero cells. The titer of Rac1-WT cells was considered as 100%. Experiments were performed in duplicate for at least three independent experiments.

Rac1 is one of the best characterized GTPases and has been implicated in negative regulation of clathrin-mediated endocytosis. To determine the Rac1 GTPase activity during DV2 entry into ECV304 cells, cell lysates from early time points p.i. were used in GST-CRIB pull-down assay. There is no obvious change in amount of total Rac1 during 1 h p.i. In contrast, Rac1 GTPase activity decreased as early as 15 min p.i. (0.3-fold) and the lowest value (0.2-fold) was seen at 30 min p.i. Thereafter, it showed a little recovery tendency with about 0.3-fold at 60 min p.i. (Fig. 2A).

In the dominant negative Rac1-N17 and Rac1-V12N17 mutants, substitution of threonine 17 for asparagines behaved as a dominant inhibitor of endogenous Rac1 function. To study the effect of dominant-negative Rac1 on virus entry, we established ECV304 cell lines expressing WT, V12N17, and N17 forms of Rac1. The cells were infected with DV2 for 1 hour and intracellular viral titers were measured. As shown in Fig. 2B, as compared with WT cells, entry of DV2 increased about 62% in cells expressing Rac1-N17 and 105% in cells expressing Rac1-V12N17, respectively. This confirms that Rac1- GTPase functions as a negative regulator for DV2 entry.

### 3. DV2 release is inhibited by treatment with actin inhibitors

**Figure 3:**
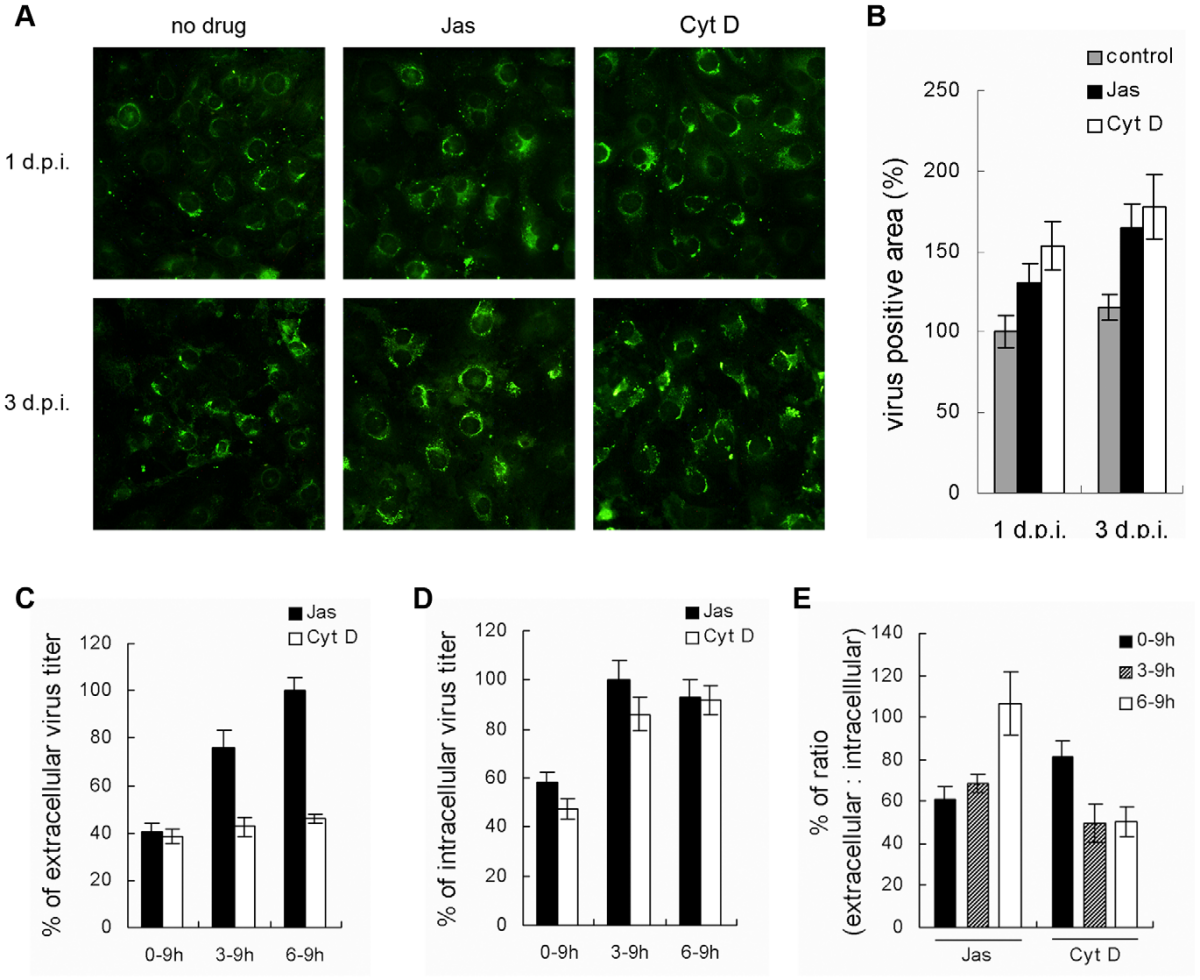
Actin is required for DV2 production and release. (A, B) Accumulation of DV2 antigens in ECV304 cells continuously treated with actin inhibitors. ECV304 cells were infected with DV2 at 37°C for 1 h. Then cells were incubated with DMEM containing Cyt D (2 µM), Jas (100 nM), or DMSO and fixed at indicated time points. The cells were labeled with mouse anti-DV2 PAb, followed by FITC-conjugated anti-mouse IgG (A). (×400) Then the relative virus positive area was measured and the area of control cells was considered as 100% (B). (C, D, E) Time course of actin inhibitors effects on DV2 infection. Medium containing 0.1% DMSO, 100 nM Jas or 2 µM Cyt D was added to the ECV304 cells at the indicated time points post infection. The supernatant and cell samples were collected at 9 h p. i., and viral titers were measured by plaque assay (C and D). The numbers under each bar represent the treatment time of Jas or Cyt D. The ratio of extra- to intracellular viral titer is shown in percents (E). Experiments were performed in duplicate for at least three independent experiments.

DV2 has previously been shown to egress by budding at the plasma membrane of infected cells, but relatively little is known about the mechanism involved in this mode of release. To access the effect of interrupted actin cytoskeleton on virus release, 2 µM Cyt D or 100 nM Jas was added at the initiation of DV2 infection and distributions of viral proteins were detected at 24 and 72 h p.i. by immunostaining. In mock-treated cells, viral antigens were generally localized at perinuclear area. While in drug-treated cells, more viral antigens accumulated throughout the cytoplasm at 24 h p.i. and the accumulation became more significantly at 72 h p.i. (Fig. 3A and 3B). The observation suggested that disturbing of actin network could inhibit the release of DV2.

Additionally, cells were treated with the two drugs (2 µM Cyt D or 100 nM Jas) from different time points post infection, and the virus titers in the medium and cell fraction were determined 9 h p.i. and were plotted as percentages of the titer in the supernatant or cell fraction of mock-treatment cells. As shown in Fig. 3C and 3D, both Cyt D and Jas could induce the reduction of supernatant viral titer, and more obvious reduction was seen in case of treatment with Cyt D (Fig. 3C). Interestingly, there was a drastic decrease in intracellular virus titers at treatment with both drugs from 0 h p.i., but only a little decrease at treatment from 3 and 6 h p.i. (Fig. 3D).

To confirm that the reduction was due to an inhibition of virus release, the ratios of extra- to intracellular infectious particles (Fig. 3E) were determined. Treatments with Cyt D at various time periods all reduced ratios significantly. In Cyt D-treated cells, the ratio was reduced to 81% when treated from 0 h p.i.; then the ratio was down to about 50% when treated from both 3 h p.i. and 6 h p.i. In contrast, treatments with Jas induce the reduction of ratio in a time-dependent manner. In Jas-treated cells, the ratio was reduced to 61% when treated from 0 h p.i.; the ratio gradually recovered to 68.6% when treated from 3 h p.i., and to about 100% when treated from 6 h p.i. As the decreased ratios indicated that the drugs have more inhibitory effects on extracellular viral titer, in combination with the result of immunostaining, the data showed that disturbing actin cytoskeleton partially blocked DV2 release. Furthermore, Cyt D showed stronger effects than Jas, suggesting that DV2 release might depend more on actin treadmilling than stabled actin filaments.

### 4. Impacts of Rac1 activity on DV2 infection

**Figure 4:**
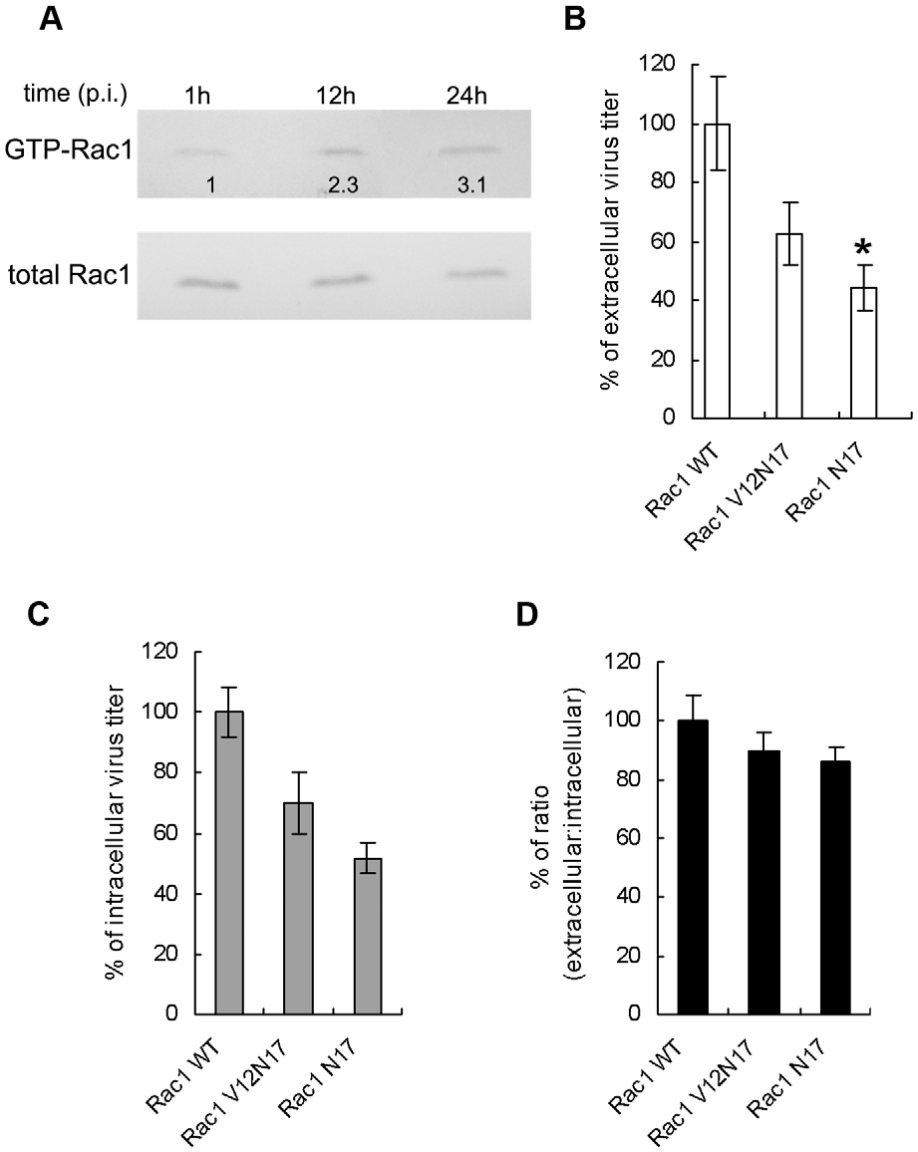
Reduced Rac1 activity inhibited DV2 production. A. Activity of Rac1 increased during DV2 infection. ECV304 cells were seeded and serum starved for about 24 h. Then cells were infected with DV2. Equal amounts of cell lysates at different time points (1 h, 12 h, and 24 h p.i.) were used to compare the GTP-bound forms of Rac1 GTPase by GST-CRIB pull-down assay. Bound GTP-Rac1 (top panels) was analyzed by SDS–12% PAGE and immunoblotted with anti-Rac1 MAb. The bottom panels show normalized cell lysates analyzed for total Rac1 as a loading control. (B, C, D,) Effects of reduced Rac1 activity on DV2 infection. ECV304 cells were stablely transfected with wild-type or dominant negative forms of Rac1. Then supernatant (B) and cell samples (C) were collected at 24 h p. i. The titers and ratio of extra- to intracellular viral titer in Rac1-WT cells was considered as 100%. Viral titers and the ratio (D) are shown in percents. Experiments were performed in duplicate for at least three independent experiments. *P<0.05 vs. wild-type Rac1.

To determine the Rac1 GTPase activity during DV2 infection, cell lysates from 1, 12, 24 h p.i. were used in GST-CRIB pull-down assay. As shown in Fig. 4A, Rac1-GTPase activity increased gradually with the progression of infection, from 2.3 fold at 12 h p.i. to 3.1 fold at 24 h p.i. Meanwhile, there was no obvious change in the total cellular Rac1 level after DV2 infection. This indicated the steady-state level of endogenous Rac1 in these cells and thus demonstrated DV2 infection induced the activation of preexisting endogenous Rac1.

To investigate the effect of dominant-negative Rac1 on DV2 infection, the cells expressing WT, V12N17, N17 forms of Rac1 were infected with DV2 for 1 hour, then supernatant and cellular samples were collected at 24 h p.i. for viral titration. As compared with Rac1-WT cells, viral titers in supernatant decreased to about 60% and 45% in cells expressing Rac1-V12N17 and Rac1-N17, respectively (Fig. 4B). Meanwhile, the intracellular virus titers were decreased to 70% around in cells expressing Rac1-V12N17 and to about 50% in cells expressing Rac1-N17 (Fig. 4C). Generally, the results indicated that suppression of Rac1 inhibited both extra- and intracellular titers of DV2 significantly. Meanwhile, we found that the dominant forms of Rac1 only led to slight reduction in ratios of extra- to intracellular infectious particles. The ratio was about 90% and 85% in cells expressing Rac1-V12N17 and Rac1-N17, respectively (Fig. 4D). Together, the results indicated Rac1 GTPase has some influence on the virus production or assembly, but little effect on DV2 release.

### 5. DV2 infection and expression of E proteins induced Rac1 activation

**Figure 5:**
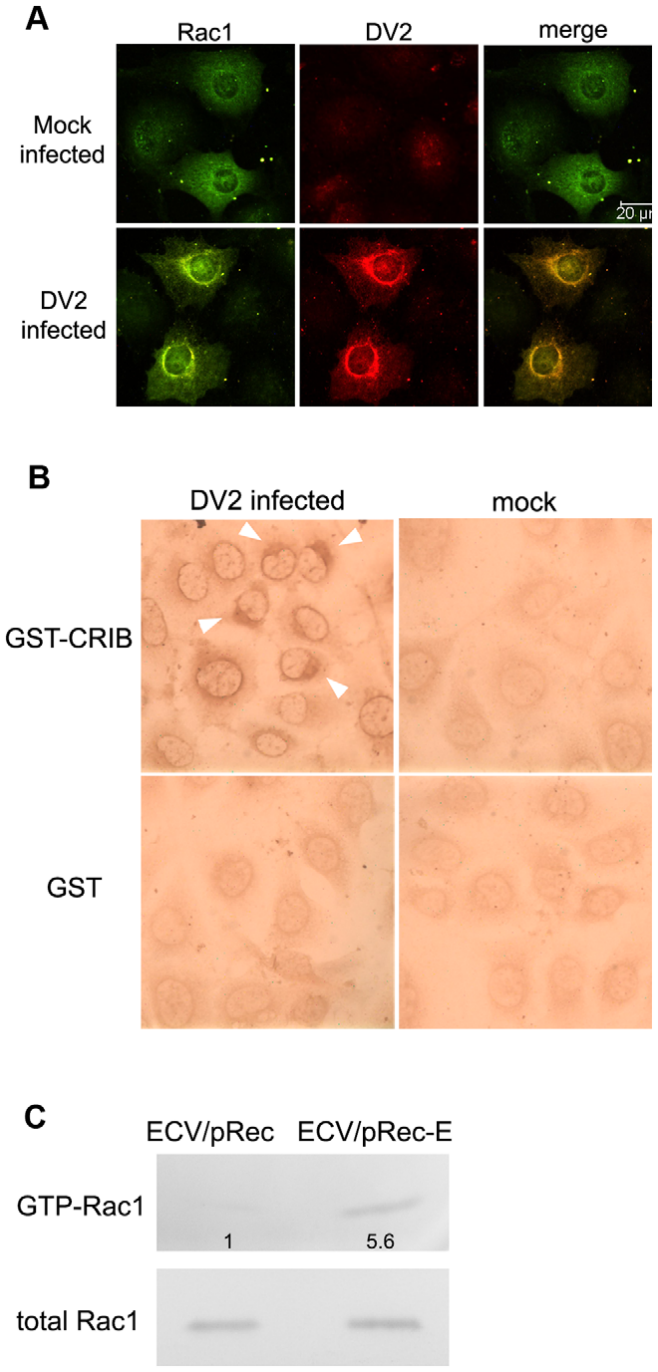
DV2 E proteins induced an increase of Rac1 activity. A. Colocalization of DV2 E proteins with Rac1 in infected cells. ECV304 cells were mock-infected or infected with DV2 and incubated for 24 h, washed, fixed in 4% paraformaldehyde for 15 min, and then permeabilized with 0.2% Triton X-100 for 5 min. Cells incubated with anti-Rac1 and a mixture of MAbs 301 and 504 were stained with FITC and TRITC-labeled secondary antibodies for Rac1 and DV2 E, respectively (Scale bars, 20 µm). B. GTP-Rac1 was detected in infected cells in situ. Briefly, cells were fixed and incubated with GST–CRIB (100 µg/ml) on ice, then cells were fixed again and GST–CRIB was detected with an anti-GST antibody and a secondary antibody conjugated with HRP. Arrowhead indicated that Rac1 GTPase concentrated in peri-nuclear region of infected cells. (×400). C. Stable expression of E protein in ECV304 cells induced an increase in Rac1-GTPase activity. ECV-pRec cells and ECV-pRec-E cells were seeded and serum starved for about 24 h. Then cells were washed twice in ice-cold PBS and lysed in 500 µl of lysis buffer. The lysates were clarified, normalized to equal amounts of total proteins, and incubated with glutathione beads containing bound GST-CRIB for 90 min at 4°C. Bound GTP-Rac1 (top panels) and total Rac1 (bottom panels) were resolved by SDS–12% PAGE and immunoblotted with anti-Rac1 MAb.

To gain insights into the mechanism by which activity of Rac1 GTPase is modulated in DV2 infection, we analyzed the distributions of Rac1 and viral envelope proteins first. Rac1 showed a diffused distribution throughout the cytoplasm in mock-infected cells. Compared with that, an enrichment of Rac1 was observed in perinuclear region of infected cells at 24 h p.i., and highly colocalized with E proteins at the same area (Fig. 5A). Accordingly, in-situ detection assay for Rac1 GTP showed that Rac1 is activated in the perinuclear region of infected cells at 24 h p.i. (Fig. 5B), whereas the activated Rac1 was rarely observed in mock-infected cells.

The activation of Rac1 by E protein was further confirmed by pull-down assays in cell lines stable-transfected with pRec or pRec-E. Cells were grown for 48 h to confluence and then cellular samples were collected for pull-down assays. As compared with ECV/pRec cells, expression of E protein in ECV/pRec-E cells induced an increase of about 5-fold in Rac1 GTPase activity (Fig. 5C). In combination with the close colocalization of E protein and Rac1, these data indicated that DV2 E protein the may trigger the activation of endogenous Rac1, which may involve in the rearrangements of actin filaments induced by DV2 infection.

### 6. DV2 E proteins interacted with actin in infected cells

**Figure 6:**
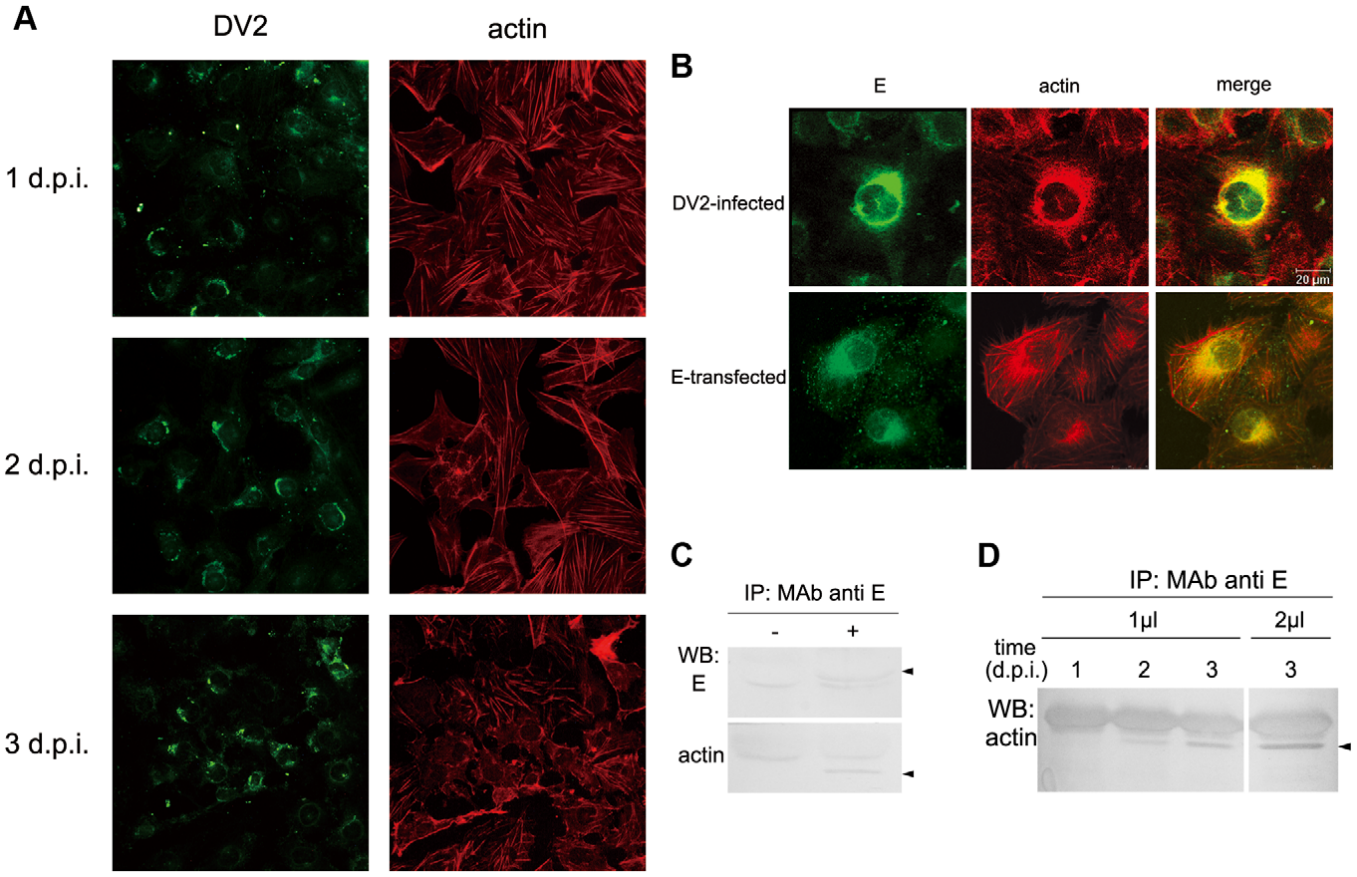
The association of DV2 E proteins with actin in infected cells. A. Reorganizations of actin cytoskeleton induced by DV2 infection. ECV304 cells were infected with DV2 at 37°C for 1 h, and fixed at different time points (1, 2, and 3 d p.i.). Then cells were labeled with mouse anti-DV2 PAb and TRITC-phlloidin, followed by FITC-conjugated anti-mouse IgG. ×400. B. Actin filaments are co-localized with E protein in DV2-infected and E-transfected ECV304 cells. The cells were fixed at 24 h p.i. or 48 h post transfection, and double-labeled with a mixture of MAbs 301 and 504, followed by FITC-conjugated anti-mouse IgG (left panels), and phalloidin-TRITC (middle panels). Merged images are given in the right panels. (Bar 20 µm). C. Co-IP of DV2 E protein with actin filaments. At 3 d p.i., cell lysate were immunoprecipitated with anti-E antibodies (a mixture of MAbs 301 and 504) followed by SDS–PAGE and immunoblot analysis with anti actin PAb or anti DV2 PAb separately. Arrowheads on the right indicate DV2 E protein (top) and actin (bottom). D. The amount of E protein immunoprecipitated with anti-actin antibodies was increased with the progression of infection. Co-IP assays were carried out with 1 µl of a mixture of MAbs 301 and 504 at 1, 2, and 3 d p.i., or with 2 µl of a mixture of MAbs 301 and 504 at 3 d p.i. The immunoprecipitated actin was detected with anti actin PAb.

To clarify whether E proteins induced actin rearrangements during DV2 infection, actin rearrangements and distribution of DV2 E proteins were detected with phalloidin–TRITC and anti-E MAb, and further analyzed with confocal immunofluorescent assay. At 1 day p.i., actin rearrangement was seen in viral antigen-positive cells. With infection progress, the actin disorganizations became more evident at 3 day p.i., when most stress fibers were disrupted and F-actin was present significantly in the periphery ruffles of cells (Fig. 6A). Actin filaments were colocalized with DV2 E protein in the perinuclear region of infected ECV304 cells. Accordingly, reorganized actin filaments also highly colocalized with transiently expressed E proteins in transfected ECV304 cells with pRec-E (Fig. 6B). This indicated the possible interaction between E protein and actin microfilaments during DV2 infection.

To further confirm the interaction between them, co-IPs were carried out to examine lysates of DV2-infected ECV304 cells. Cell lysates were immunoprecipitated with anti-E antibodies followed by SDS-PAGE and immunoblot analysis with anti-actin antibody. An actin-E protein complex could be co-immunoprecipitated using anti-E antibodies (Fig. 6C). Co-IP was also performed with DV2- infected suckling mouse brain, and similar result was obtained (data not shown). The reverse immunoprecipitation was performed using anti-actin antibodies followed by immunoblot analysis with anti-E antibodies. However, the association of E with actin could not be observed. This may be due to the fact that the cytoplasma is very rich in actin filaments, so that the loading quantity of 10 µg per lane is below the detection level of E protein. In the subsequent co-IPs, we used anti-E antibodies to first carry out the immunoprecipitation. With the progression of infection, the amount of actin associated with viral E proteins was increased and peaked at 3 d p.i. (Fig. 6D). The results strongly suggested the close association of E proteins with the actin filaments. Taken together, these data indicated E is an important viral component in the signal pathway triggering actin rearrangements during the infection.

## Discussion

### Actin cytoskeleton is required for DV2 infection

Flaviviruses enter the host cells by receptor-mediated endocytosis. DC-SIGN (dendritic-cell-specific ICAM-grabbing non-integrin), GRP78/BiP (glucose-regulating protein 78), and CD14-associated molecules have been suggested as primary receptors for DV [28]–[31]. We and other groups showed that β3 integrin, a prominent endothelial cell receptor, is identified as the functional receptor necessary for WNV and DV2 entry into vertebrate cells [5]. Subsequent to the initial interaction of ligand/virus with the cellular receptor, an internalization signal is usually triggered to facilitate the endocytosis of the virus into cells.

DV, as well as WNV, particles enter mammalian and mosquito cells by clathrin-dependent endocytosis [14], [15], [17], [32], Actin cytoskeleton is critical for clathrin-coated structure dynamics and macropinocytosis [27]. In this study, significant repolymerizations of actin cytoskeleton at early infection were observed, and disruption of actin filaments with Jas or Cyt D inhibited significantly the entry process of DV2 in a time and dose dependent manner. Our results are consistent with recent reports in which, it was found that inhibition of macropinocytosis with Cyt D caused a reduction in DV2 entry, and the inhibition effects of Cyt D depend upon the serotype of the DV to some extent [16], [17]. All together, these results suggested that DV2 may manipulates actin cytoskeleton and the associated signaling cascades to facilitate its entry.

Cytoskeleton is also believed to be play active roles in viral intracellular transport, maturation and release. Previously, we observed that both vimentin and microtubule were reorganized in DV2 infection [33], and role for actin in the release of WNV has been reported by others [11]. Here, we also observed significant repolymerizations of actin cytoskeleton during DV2 infection and accumulation of large amount of DV2 antigens in cells treated by two actin inhibitors, indicating that actin also involved in DV2 release. However, there was a significant difference in inhibition potency between these two drugs. As drug incubation time decreased, the ratio of extra- to intracellular virus in cells treated with Jas gradually recover to the control level, whereas the ratios of cells treated with Cyt D kept down to 50%. The findings are very interesting because these two inhibitors destabilize actin dynamics through different mechanisms: Cyt D inhibits the polymerization of subunits by binding to the plus-ends of the actin filaments, while Jas, a potent inducer of actin polymerization, enhances actin stabilization by inhibiting the depolymerization of actin filaments. The decrease of extracellular DV2 yields could be resulted from the inhibition of the growing actin barbed ends, which are essential for generating the vectorial force for membrane bending and virus budding. Our findings indicated that the availability of monomeric actin to form newly filamentous actin is essential for DV2 release. In other words, DV2 release might depend more on actin treadmilling than stabled actin. In combination with WNV results reported by others, it is speculated that utilizing actin may be a common mechanism for flaviviruses release.

Next, we are interested whether E protein, which is an important viral structural protein and serves as viral adsorption protein, involve in the interaction of DV2 and actin. In this study, the high co-localization of DV2 E protein with actin was observed by confocal microscopy analysis, and their interaction was further confirmed by co-IP assay. Moreover, both the amount of actin associating with E proteins (Fig. 6A) and the level of reorganization of actin cytoskeleton (Fig. 6D) are increased with the progression of infection. These indicated the close association of DV2 E protein with actin. Our results are consistent with a recent study reported by Chu [12], who found that actin is associated with protein C from 6 h p.i. and then with protein E from 10 h p.i. in WNV infected cells, suggesting that the association between actin and viral structural proteins may be common in flaviviruses. Moreover, previous studies have demonstrated that rearrangements of actin filaments and overexpression of integrinβ3 induced by DV2 in endothelial cells may contribute to the increase in vascular permeability observed in DHF/DSS [5]–[7]. Gelatinolytic matrix metalloproteinase-9 and matrix metalloproteinase-2 overproduced by DV2-infected immature dendritic cells down-regulate the expression levels of cell adhesion molecules, induce the redistribution of the actin filaments, and enhance endothelial permeability [34]. All together, it was speculated that a direct effect of viral protein may also contribute to the structural modifications of actin cytoskeleton in infected cells, and to the increased permeability of the endothelial cells during DV infection, especially when large amounts of virons are produced and released.

### The involvement of Rac1 GTPase in DV2 infection

The negative regulation of clathrin-mediated endocytosis of cell surface receptors mediated by Rac1 has been implicated in both polarized and non-polarized cells. In MDCK cells, Rac1V12 expression decreased the rates of apical and basolateral endocytosis [35]. In Hela cells, overexpression of constitutively active forms of Rac1 causes inhibition of transferrin and EGF receptor internalization [18], [26]. One mechanism by which Rac1 might participate in clathrin-mediated endocytosis is the modulation of phosphatidylinositol (PI) lipid metabolism, which plays a key role in this process [36]. Rac1 has been shown to interact with a number of enzymes that regulate PI metabolism, including type I PI 3 -kinase and synaptojanin 2 [37], [38]. Recently, a guanine nucleotide exchange factor splice variant designated Ost-III is identified as a regulator of Rac1 involved in the inhibition of receptor endocytosis [26].

In this study, we observed that Rac1 GTPase activity decreased at the early stage of DV infection. The role of Rac1 GTPase in regulating the DV2 infection was clearly demonstrated by the increase of virus entry in cells expressing Rac1-V12N17 or Rac1-N17. The results suggested that the initial mediator of transient Rac1 inactivation may be viral receptors in response to DV2 binding and/or internalization. This observation was supported by an earlier study showing that GTPase Rab 5, which regulates transport Rac1 to early endosomes, was essential for cellular entry of both WNV and DV [14]. Since the Rac1 dominant negative mutants show a reduced affinity for nucleotides, the increased DV2 entry observed may be due to the abrogation of downstream signaling of active Rac1.

It is noteworthy that Rac1 activity is up-regulated in the late phase of DV2 infection, and dominant negative Rac1 inhibited DV2 production and release. Further study showed that Rac1 is activated and colocalized with E protein at perinuclear area in DV2- infected cells, and Rac1 activity increased in cells expressing E protein. These results raise the possibility that Rac1 activated by viral protein is involved in the reorganization of actin cytoskeleton after DV2 infection. Rac1 has been implicated in actin nucleation and membrane ruffling. Rac1 and the adapter protein Nck activate actin nucleation through WAVE1 (WASP -family verprolin homologous protein), PAK and Arp2/3 complex [39], [40]. A recent study revealed that there is also a critical function for Rac1 in tight junction assembly. Tight junction assembly was disrupted profoundly in mammalian epithelial cells depleted of Par-3 while Rac1 was constitutively activated, and the assembly of tight junctions is efficiently restored by a dominant-negative Rac1 mutant [41]. These studies support the hypothesis that activated endogenous Rac1 contributes to the rearrangements of actin filaments and intercellular junctions at later phase of DV infection.

The dysfunction of vascular endothelium and increased vascular permeability is the hallmark of severe DV infection in humans. Previous studies showed that the phenomenon of antibody-dependent enhancement plays an important role in the immunopathogenic aspect of DHF. In secondary heterotypic infection, pre-existing, non-neutralizing antibody induces virus-antibody complex binding to Fc-receptor bearing cells, such as dentritic cells and monocytes/macrophages, and results in increased T-cell activation as well as higher serum cytokine levels. Then the virus escapes and infects greater numbers of cells which can lead to a greater viral load. High dengue viremia during dengue fever or viremia detected after defervescence have been shown to associate with development of DHF [42], [43]. Vascular endothelium, a barrier between blood and tissue, will directly interact with the viral particles and virus-antibody complexes during the viremia. Dengue antigens have been demonstrated in biopsies of lung, liver and brain vascular endothelium of patients [44]–[46]. In this study, our results showed a possible correlation between the accumulation of virus envelope proteins and the endothelium dysfunction, which could be a mechanism involved in the etiology of DHF/DSS.

Taken together, the results demonstrated that dynamic treadmilling of actin is necessary for DV2 entry, production and release, and Rac1 GTPase also plays critical roles in those processes. We also confirmed the interaction between E protein and actin, which indicated a direct effect of viral protein on the structural modifications of actin cytoskeleton. Further understanding of the various components of signal cascades manipulated by DV would lead to a better understanding of DV interactions with host cells and their outcomes, all of which would eventually lead to the development of better control measures against DV infection.
